# Transcriptomic profiling of diabetic retinopathy: insights into RPL11 and bisphenol A

**DOI:** 10.3389/fendo.2025.1705233

**Published:** 2025-11-26

**Authors:** Jian Zhang, Xin Yang

**Affiliations:** 1 Department of Ophthalmology, People’s Hospital of Tongchuan, Tongchuan, Shaanxi, China; 2 Shaanxi Eye Hospital, Xi’an People’s Hospital (Xi’an Fourth Hospital), Affiliated People’s Hospital of Northwest University, Xi’an, Shaanxi, China

**Keywords:** diabetic retinopathy, ribosomal protein L11 (RPL11), bisphenol A (BPA), molecular docking, molecular dynamics

## Abstract

**Background:**

Diabetic retinopathy (DR) is a leading microvascular complication of diabetes mellitus, causing irreversible vision loss in adults worldwide. However, the molecular mechanisms underlying DR pathogenesis—especially the crosstalk between core genes, immune microenvironment, and environmental factors remains incompletely elucidated. This knowledge gap hinders the development of effective preventive and therapeutic strategies for DR, making it urgent to identify key molecular targets and regulatory pathways.

**Objective:**

To elucidate the molecular mechanisms underlying DR through transcriptomic analysis, and explore the potential interaction between ribosomal protein L11 (RPL11) and bisphenol A (BPA) using in silico approaches.

**Methods:**

The gene expression dataset associated with DR (GSE221521, Platform: GPL24676) was preprocessed and statistically evaluated via R (version 4.5.1). Differentially expressed genes (DEGs) were identified using linear models with empirical Bayes moderation (limma R package, version 3.65.7), and weighted gene co-expression network analysis (WGCNA) was applied via the WGCNA R package (version 1.73) to detect co-expressed gene modules. Functional annotations were performed via Gene Ontology (GO) and Kyoto Encyclopedia of Genes and Genomes (KEGG) analyses (clusterProfiler R package, version 4.17.0). To validate the core gene, we conducted Gene Set Enrichment Analysis (GSEA, fgsea R package, version 1.35.8), immune cell infiltration profiling (CIBERSORT algorithm, version 1.03), molecular docking (AutoDock Vina, version 1.2.0), and molecular dynamics simulations (GROMACS, version 2022.4).

**Results:**

Differential expression analysis (thresholds: |log_2_-fold change (FC)| ≥ 0.585 [1.5-fold change] and Benjamini–Hochberg (BH)-adjusted P < 0.05) identified 341 DR-specific DEGs (intersection of DEGs from DR vs. healthy controls [Nor] and DR vs. diabetes mellitus [DM] without retinopathy). Additionally, WGCNA (soft threshold power β=3, scale-free R²=0.8) identified 38 co-expressed gene modules, with the “black and brown” modules showing the strongest correlation with DR (Spearman correlation coefficient > 0.6, adjusted P < 0.001). Venn analysis of 341 DR-specific DEGs and WGCNA core genes (gene significance [GS] > 0.5, module membership [MM] > 0.8) revealed 201 co-expressed genes, and GO and KEGG pathway enrichment analyses were performed (P < 0.05). RPL11 was identified as a core gene with high diagnostic potential in peripheral blood (area under the curve [AUC] = 0.796, 95% Confidence Interval (CI):0.716-0.875), with significantly downregulated expression (log_2_FC = -0.67, adjusted P = 4.19×10^-5^) observed in the DR cohort. It also exhibited significant binding affinity with BPA in molecular docking simulations (binding energy = -5.491 kcal/mol, and molecular dynamics simulations confirmed the BPA-RPL11 complex’s stability (backbone RMSD: 0.45–0.55 nm after 60 ns, persistent hydrogen bonds: 2–5 throughout the simulation), providing hypothesis-generating clues for DR-related molecular research.

**Conclusion:**

This research analyzed molecular associations related to DR using peripheral blood transcriptomic data, identifying RPL11 as a hypothesis-generating molecule with potential associations with DR in peripheral blood—this finding serves as a hypothesis-generating candidate for subsequent DR-related molecular research. Environmental BPA exposure was found to be associated with RPL11 dysregulation in peripheral blood (in silico evidence: BPA-RPL11 specific binding and stable complex formation), suggesting a potential correlative link to DR progression that requires further empirical validation. These findings highlight the need for additional research to explore the possibility of minimizing BPA contamination as a potential DR risk mitigation strategy, rooted in hypothesis-generating insights.

## Introduction

Diabetic retinopathy (DR), a microvascular complication of diabetes mellitus, represents the leading cause of preventable blindness in working-age adults globally. Epidemiological studies have shown that ~35% of diabetic patients develop DR, with >10% progressing to vision-threatening stages, causing substantial socioeconomic burdens through permanent visual disability (1). Pathogenesis involves a complex interplay of metabolic, inflammatory, and neurovascular disturbances. Early detection remains challenging due to subclinical progression, with ~50% of cases undiagnosed until advanced stages. Current invasive interventions (laser photocoagulation or intravitreal anti-VEGF therapies) carry risks of retinal scarring and ocular hypertension (2, 3).

Bisphenol A (BPA), a pervasive endocrine-disrupting chemical, infiltrates humans via dietary packaging, thermal paper, and contaminated water. Despite widespread exposure and its established role in disrupting glucose homeostasis [impairing insulin sensitivity and β-cell function (4)], the contribution of BPA to diabetic microvascular complications remains largely uncharacterized (5).

Prior studies have linked ribosomal protein dysregulation—especially RPL11—to metabolic disorders: RPL11 downregulation in pancreatic β-cells impairs insulin secretion (6), while ribosomal stress in endothelial cells exacerbates diabetic macroangiopathy (7). Additionally, BPA has been shown to disrupt protein–protein interactions in ribosomal complexes in non-ocular cell types (8), providing a rationale for exploring whether BPA-induced RPL11 dysregulation contributes to DR pathogenesis.

This study systematically evaluated the pathogenic role of BPA in DR by integrating Comparative Toxicogenomics Database (CTD)-driven bioinformatics with molecular docking simulations. We hypothesize that BPA may induce ribosomal protein L11 (RPL11) dysregulation, potentially triggering ribosomal stress, impaired protein synthesis, and vascular injury. By mapping the proteome-wide interactions of BPA in DR, this approach bridges computational efficiency with mechanistic insight to address critical gaps in environmental ophthalmology. Our objectives are as follows:

① Identification of molecules in peripheral blood showing associations with DR, serving as hypothesis-generating candidates for potential chemopreventive targets.
② Discovery of noninvasive peripheral blood biomarkers for exploring environmental trigger associations with DR.
③ Establishing hypothesis-generating predictive models for exploring chemical-related ocular toxicity in DR.

## Materials and methods

**Figure 1:**
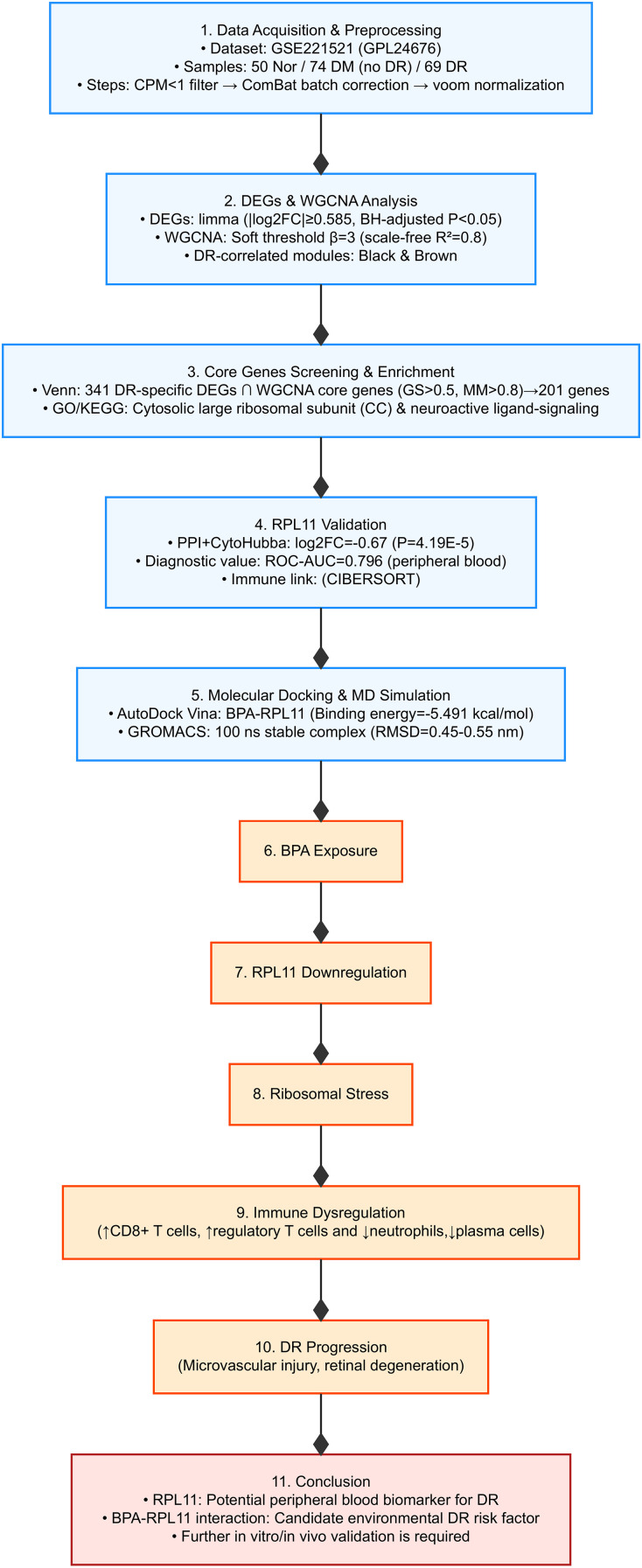
Overview of this study.

The overview design of our work was shown in **Figure 1**.

### Ethical

This study employed publicly available transcriptomic datasets retrieved from the Gene Expression Omnibus (GEO) database. The secondary analysis of these preexisting datasets was conducted in accordance with international ethical guidelines governing the reuse of anonymized genomic data, as stipulated by the ICMJE recommendations and FAIR (Findable, Accessible, Interoperable, Reusable) principles. This approach qualifies for ethical exemption, as it involves no new human subject involvement or identifiable private information.

### Data acquisition and preprocessing

Public transcriptomic datasets were retrieved from the GEO repository (Accession: GSE221521; Platform: GPL24676) via the search term “Diabetic Retinopathy”. The selection criteria were as follows:

Dataset type: RNA sequencing (RNA-seq) expression profiling
Species: Homo sapiens
Study design: Case–control comparisons with ≥50 samples per group
Publication period: 2022–2025

The dataset included peripheral blood RNA-seq profiles from: 50 healthy controls (Nor), 74 diabetes mellitus (DM) patients without retinopathy and 69 confirmed DR patients were included. DR diagnosis followed the International Clinical Diabetic Retinopathy Disease Severity Scale (2002 consensus).

To isolate DR-specific molecular signals from diabetes-driven changes, we conducted two sets comparisons: 1) DR vs. Nor; 2) DR vs. DM. This design allows us to distinguish alterations specific to DR from those caused by diabetes itself, while also capturing diabetes-related changes independent of retinopathy.

Data processing workflow was expanded as follows:

1. Raw gene-level counts were annotated via GPL24676 platform annotations.
2. Probe IDs were converted to gene symbols, and ambiguous probes mapping to multiple genes were removed.
3. Lowly expressed gene filtering: Genes with counts per million (CPM) < 1 in ≥ 50% of samples were excluded to ensure reliable downstream analysis.
4. Batch effect adjustment: Potential batch effects were corrected using the ComBat function from the sva R package (version 3.57.0), with batch information inferred from the GSE221521 dataset metadata to reduce technical variation.
5. Data normalization: The filtered and batch-corrected raw counts were further processed using the voom function from the limma package (version 3.65.7) to generate log_2_-transformed counts per million values.

### Differential expression analysis

Differentially expressed genes (DEGs) were identified via the limma package (version 3.65.7) in R, with rigorous adjustment for potential confounders and technical variation:

1. Data transformation: Prior to model fitting, the batch-corrected raw counts were processed using the voom function (from limma package), which converts raw counts to log_2_-counts per million (log_2_CPM) while estimating the mean-variance relationship of RNA-seq data.
2. Contrast analysis: Two independent contrasts were analyzed with consistent thresholds: |log_2_-fold change (FC)| ≥ 0.585 (equivalent to 1.5-fold change) and Benjamini–Hochberg (BH)-adjusted P < 0.05 (explicit multiple testing correction method). The contrasts included: 1) DR vs. Nor; 2) DR vs. DM.
3. Validation of batch effect correction: After model fitting, principal component analysis (PCA) was performed on the voom-transformed data to verify that batch effects were effectively reduced.
4. Visualization: ggplot2 R package (version 4.0.0) was used to generate volcano plots for each contrast, highlighting upregulated (red) and downregulated (green) DEGs. Heatmaps of the top 50 DEGs (ranked by adjusted P) for each comparison were constructed via pheatmap R package (version 1.0.13), with Z score-normalized log_2_CPM values and hierarchical clustering.

### WGCNA screening for signature and coexpressed genes

We performed WGCNA on the batch-corrected and voom-transformed log_2_CPM gene expression matrix to ensure consistency with differential expression analysis and reduce technical confounding via the WGCNA R package (version 1.73) within the R programming environment. This investigation facilitated the classification of genes into distinct modules, enabling the identification of their most significant associations with the progression of DR.

Soft threshold power (β=3): Selected via scale-free topology analysis (achieved scale-free R²=0.8), meeting the standard threshold for a reliable scale-free network.

Minimum module size: 30 genes: Balances stability and functional specificity (modules with <30 genes are often noise-driven).

Module–trait association statistics: Pearson correlations between each module’s eigengene and DR status were calculated, with Benjamini–Hochberg (BH)-adjusted P values to control false discovery rate (FDR) across multiple tests.

We selected the pivotal genes within the DR-correlated modules by applying a threshold for gene significance (GS) > 0.5 and gene–module membership (MM) > 0.8, yielding core module genes associated with DR. Subsequently, to further refine DR-specific core genes, we conducted two rounds of Venn analysis: first to screen DR-specific DEGs (excluding DM-driven changes), and second to intersect these DR-specific DEGs with the DR-correlated WGCNA core module genes.

### GO and KEGG enrichment analysis

To analyze the signaling pathways and biological functions enriched by genes (overlapping DR-specific DEGs and WGCNA core genes), we imported the selected overlapping genes into R software. We employed enriched GO (Biological Process [BP], Cellular Component [CC], Molecular Function [MF]) and KEGG algorithms via the clusterProfiler R package (version 4.17.0) to analyze the pathways linked to the genes. Statistical significance was defined as BH-adjusted P < 0.05. This study provides valuable insight into the roles of these genes in diverse biological activities.

### Construction of networks and identification of critical genes

The identified overlapping genes were imported into the STRING online database (https://string-db.org/) to construct a protein–protein interaction (PPI) network. We retrieved the pertinent data for additional analysis. The PPI data were subsequently imported into Cytoscape version 3.10.3(confidence score ≥ 0.4). Next, we employed the cytoHubba plugin to pinpoint the essential genes within the network. The 8 genes that ranked highest on the basis of their degree centrality were recognized as the principal genes.

### Identification and verification of genes associated with disease signatures

We utilized the glmnet R package (version 4.1-10) in the R programming environment to select genes via regularization methods such as LASSO regression, which helps identify important genes for disease diagnosis. Using the pROC R package (version 1.19.0.1), we created ROC curves to assess how effectively these genes differentiated between Nor and DR states (with AUC ≥ 0.74 considered robust diagnostic performance). This evaluation confirms the value of their practical application. Additionally, we utilized the rms R package (version 8.1-0) and rmda R package (version 1.6) to confirm the significance of the chosen key genes. We developed nomograms to illustrate the associations between genes and DR risk. We evaluated the model’s predictive accuracy and investigated the relationships between genes and diseases via model fitting, calibration, and validation.

### Level of immune cell infiltration

We meticulously assessed the relative proportions of various immune cell types within the RNA-seq expression profiles using CIBERSORT algorithm (version 1.03) and performed an in-depth examination of the alterations in these immune cells among patients with DR. Through a comparative analysis of the immune cell distributions between healthy subjects and individuals with DR, we observed significant discrepancies in their immune cell profiles (statistical test: Student’s t-test, P < 0.05 considered significant).

### Gene set enrichment analysis

GSEA was conducted via the fgsea R package (version 1.35.8) to elucidate pathway-level mechanisms linked to RPL11 expression dynamics in DR. Samples were stratified into high-expression (≥ 75th percentile) and low-expression (≤ 25th percentile) cohorts. Gene ranking was performed on the basis of the log_2_FC values between the two cohorts. Pathway enrichment was analyzed via GSEA with the KEGG 2023 gene set library. The parameters included 1,000 phenotype permutations, a minimum gene set size of 15, and significance thresholds of P < 0.05 and |normalized enrichment score (NES)| > 1.5.

### Molecular docking

Core gene-related candidate targets were cross-referenced against the Comparative Toxicogenomics Database (CTD; http://ctdbase.org/) to identify small molecules with literature-supported associations with ribosomal stress pathways. The compounds were prioritized via tripartite criteria: ≥3 independent experimental validations in mammalian systems, mechanistic links to ribosomal stress pathways, and structural availability in PubChem. High-resolution crystallographic structures of the target proteins were obtained from the RCSB Protein Data Bank (PDB). Ligand 3D conformers were downloaded from PubChem (https://pubchem.ncbi.nlm.nih.gov). Proteins were preprocessed in AutoDock Tools (version 1.5.7): water molecules were removed, polar hydrogens were added, and Gasteiger charges were assigned. Ligands were energy-minimized via UFF force fields. Docking simulations were performed in AutoDock Vina (version 1.2.0) with a grid box (30Å × 30Å× 30Å, 0.05 nm spacing) centered on each protein’s active site. Binding poses were ranked by the calculated binding energy. The highest-ranking complexes were subjected to 100-ns molecular dynamics simulations to evaluate their stability. PyMOL (version 2.5.2) visualized hydrogen bonding, hydrophobic interactions, and binding pocket complementarity.

### Molecular dynamics simulation

Molecular dynamics simulation was performed via GROMACS (version 2022.4) to assess the binding characteristics of the complex, with detailed parameters as follows:

The BPA-RPL11 complex was solvated in TIP3P water with Na^+^ ions to neutralize the charge. After energy minimization, the steepest descent approach was employed to optimize the molecular dynamics simulation system, which was subsequently followed by 1,000,000 steps of isothermal isovolumetric (NVT) equilibration followed by isothermal isobaric (NPT) equilibration. After these equilibrations, an unconstrained simulation was conducted, extending over a total duration of 50,000,000 steps with a timestep of 2fs, culminating in an overall simulation time of 100ns.To elucidate the binding interaction between BPA and RPL11, a series of simulations were carried out, during which the hydrogen bonds (H-bonds), solvent accessible surface area (SASA), root mean square fluctuation (RMSF), root mean square deviation (RMSD), and radius of gyration (Rg) of the RPL11-BPA complexes were meticulously calculated (9).

## Results

### Differentially expressed gene analysis

**Figure 2:**
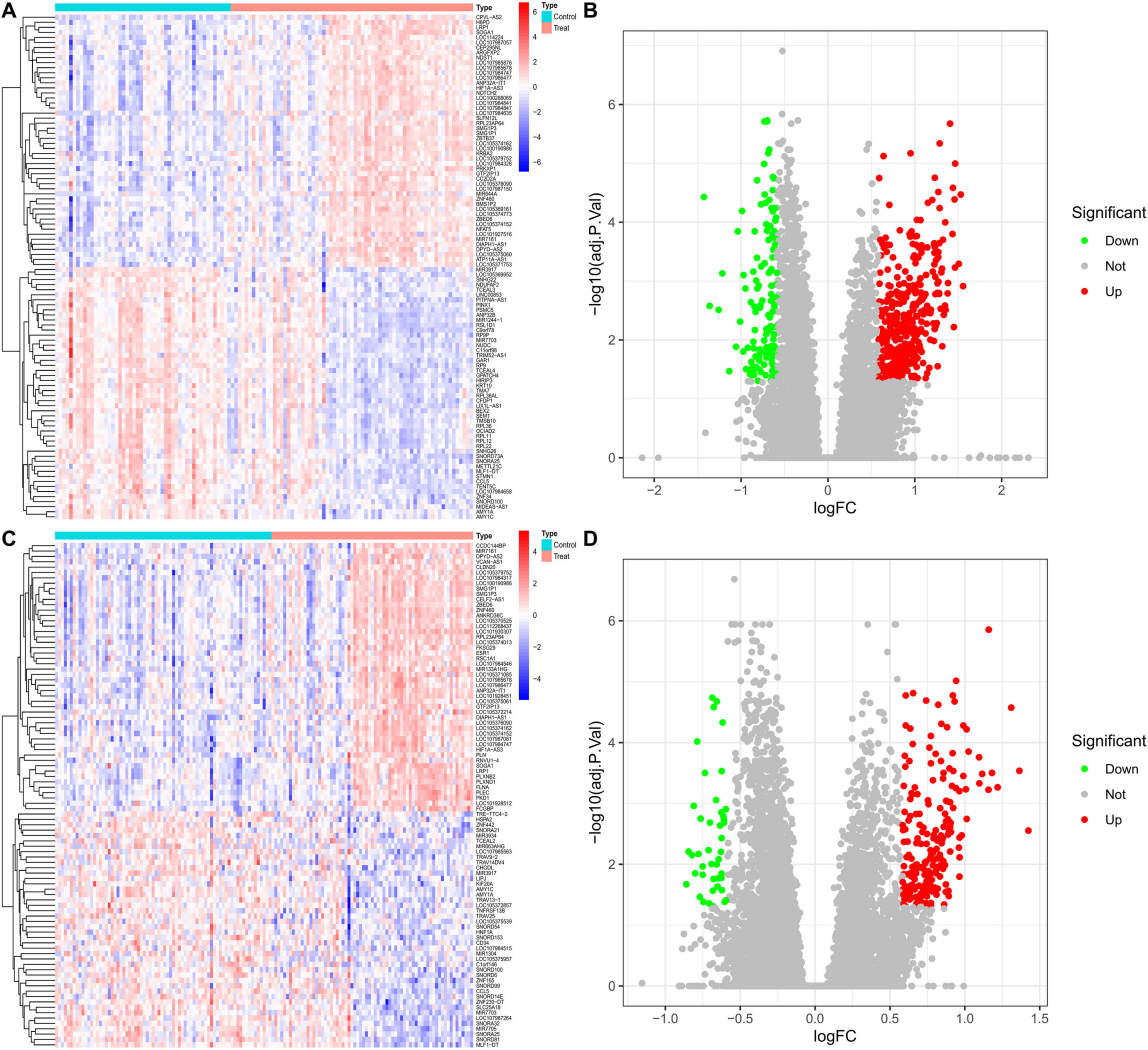
**(A)** Heatmap of DEGs for DR vs. Nor. **(B)** Volcano plot of DEGs for DR vs. Nor. **(C)** Heatmap of DEGs for DR vs. DM. **(D)** Volcano plot of DEGs for DR vs. DM.

To isolate DR-specific molecular alterations, two sets of differential expression analyses were conducted with the threshold |log_2_FC| ≥ 0.585 and BH-adjusted P < 0.05, yielding the following results: For the first contrast DR vs. Nor: A total of 509 DEGs were identified, including 380 upregulated and 129 downregulated transcripts (**Figures 2A, B**); For the second contrast DR vs. DM: 264 DEGs were detected, with 220 upregulated and 44 downregulated transcripts (**Figures 2C, D**).

### WGCNA screening module core genes

**Figure 3:**
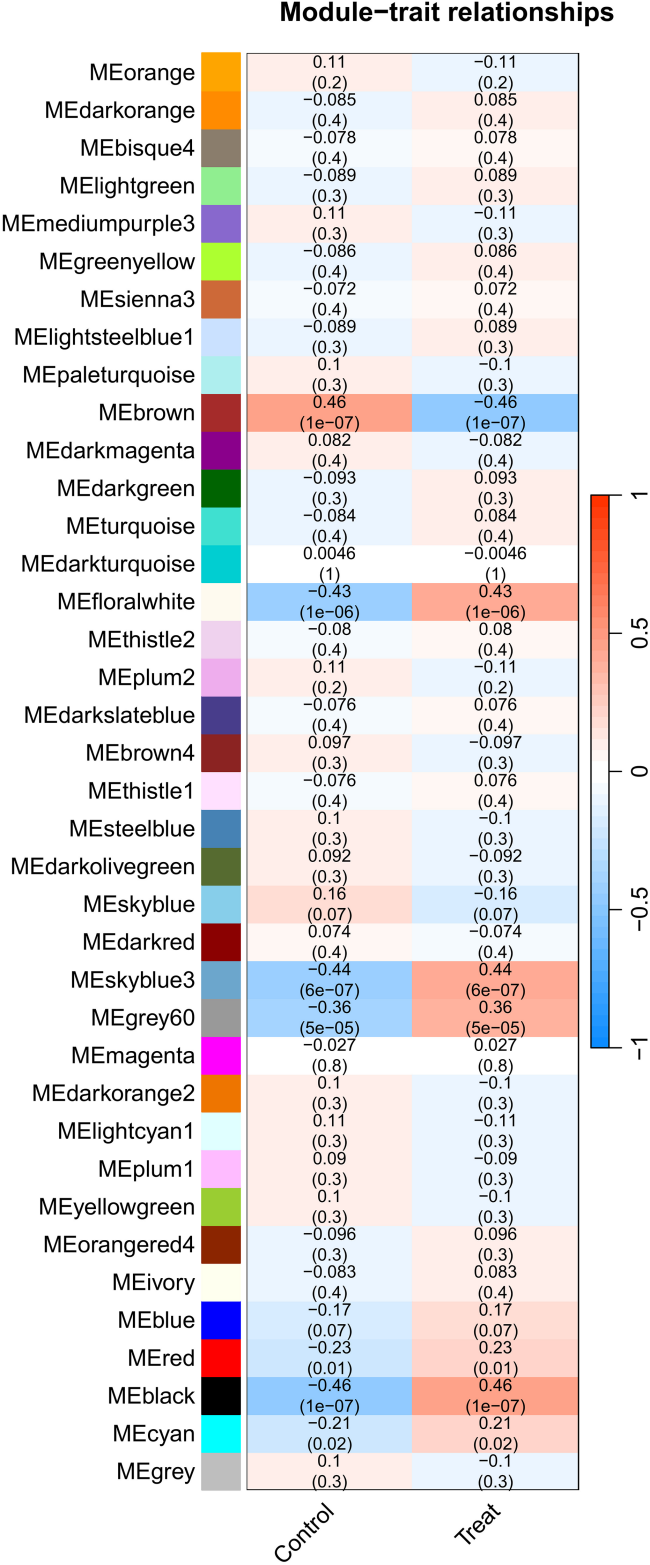
Module-trait correlation heatmap.

WGCNA with scale-free topology (soft threshold power β=3, scale-free R²=0.8) generated 38 co-expressed gene modules from the GSE221521 transcriptome (**Figure 3**). A correlation heatmap was generated to illustrate the relationships between modules and diseases, employing the Spearman correlation coefficient (**Supplementary Figures 1**, **7**). The findings revealed that the “black and brown” modules presented the strongest correlation with DR, comprising a total of 4361 genes.

### Venn analysis

**Figure 4:**
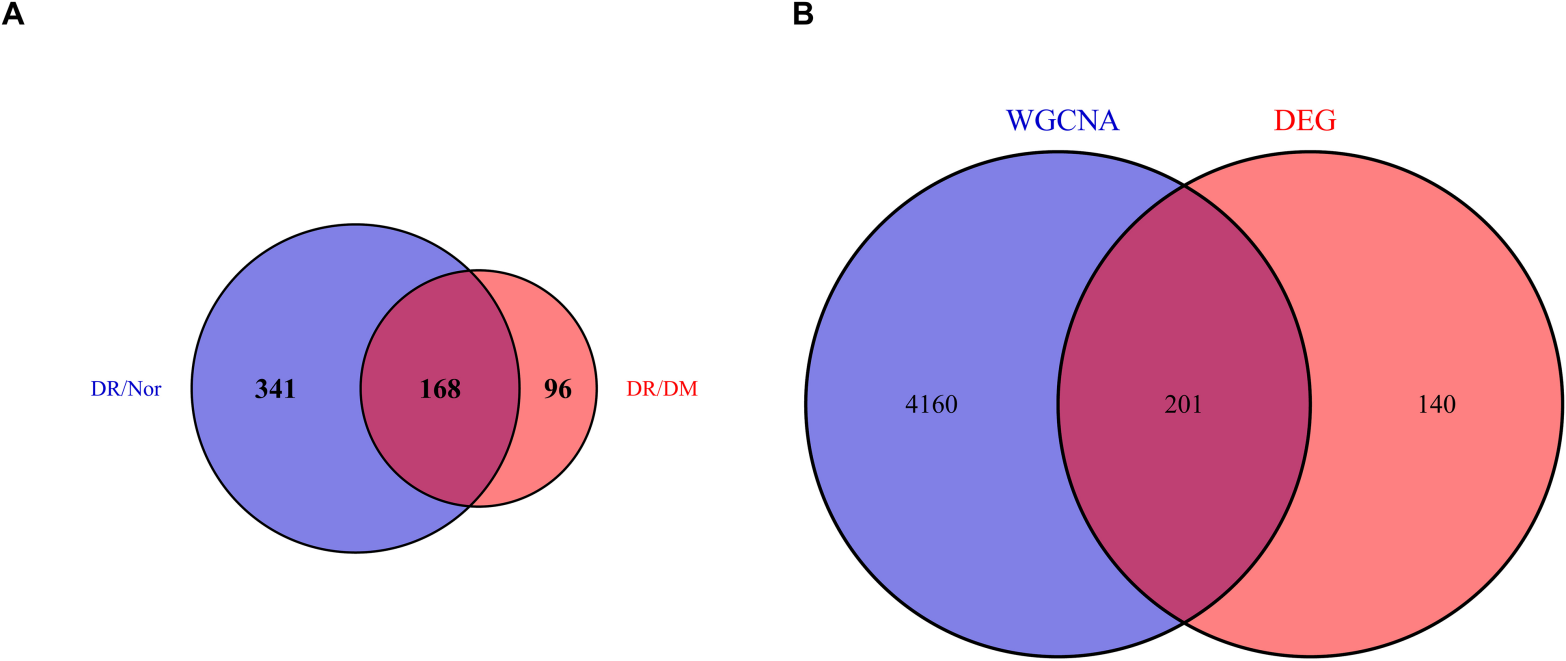
**(A)** 341DR-specific DEGs. **(B)** 201 common targets in both WGCNA and DR-specific DEG.

Two rounds of Venn analysis were performed to screen DR-specific core genes:

First round (DR-specific DEGs screening): Intersection of DEGs from DR vs. Nor (509 DEGs) and DR vs. DM (264 DEGs) yielded 341 DR-specific DEGs—these genes represent molecular changes unique to DR, excluding alterations driven by diabetes itself (**Figure 4A**).
Second round (DR key relevant genes screening): Intersection of the 341 DR-specific DEGs with the DR-correlated WGCNA core module genes (GS > 0.5, MM > 0.8; derived from the “black and brown” modules) identified 201 DR key relevant genes (**Figure 4B**). These genes integrate both DR-specific expression patterns and strong association with DR progression, serving as the core gene set for subsequent functional analyses.

### GO and KEGG analyses

**Figure 5:**
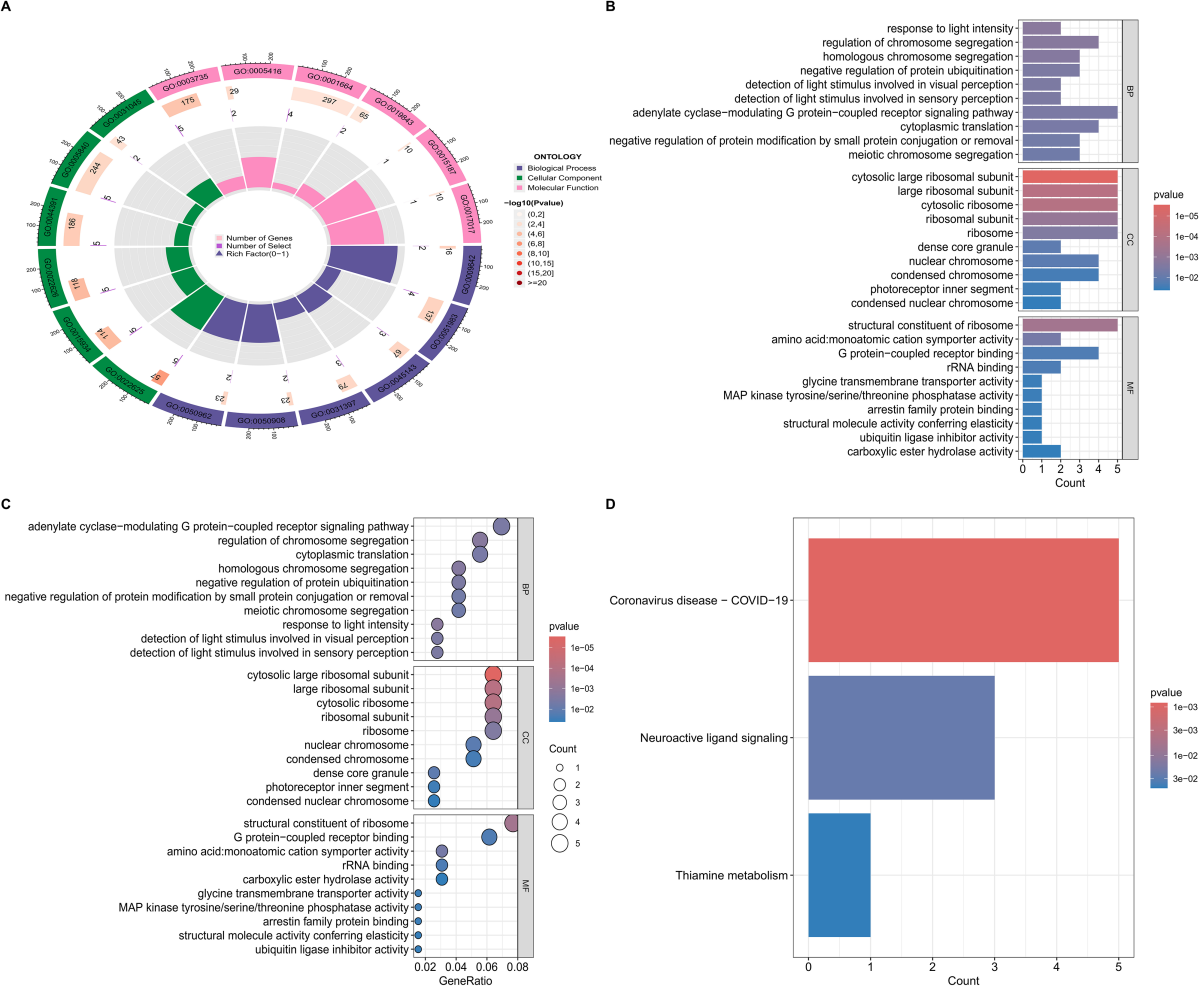
**(A)** GO enrichment analysis; Triadic histogram depicting the GO biological processes (BP), cellular components (CC) and molecular functions (MF),of core targets. In this visualization, purple denotes the BP terms, green represents the CC terms, and pink signifies the MF terms. **(B)** Bar chart of the GO enrichment analysis for the candidate hub genes. **(C)** The bubble map of the GO enrichment analysis of the candidate hub genes. **(D)** KEGG pathway enrichment analysis.

In the present investigation, data pertaining to 201 DR key relevant genes were imported into R software (version 4.5.1) and comprehensive analyses of GO and KEGG pathways were performed via the clusterProfiler R package (version 4.17.0). The GO analysis indicated that the biological process category prominently encompasses essential biological activities, particularly response to light intensity, detection of light stimulus involved in visual perception and detection of light stimulus involved in sensory perception. Furthermore, the cellular component assessment highlighted the importance of various cellular structures, notably the cytosolic large ribosomal subunit. The analysis of molecular functions emphasized critical roles, including that of a structural constituent of ribosomes as well as the G protein−coupled receptor binding (**Figures 5A–C**). Additionally, the KEGG pathway analysis revealed that the Neuroactive ligand signaling and Coronavirus disease–COVID-19 pathway were enriched predominantly in several key signaling pathways (**Figure 5D**; **Supplementary Figure 2**), which may represent DR-specific pathogenic pathways.

### Interaction networks and core genes of differential gene-encoded proteins

**Figure 6:**
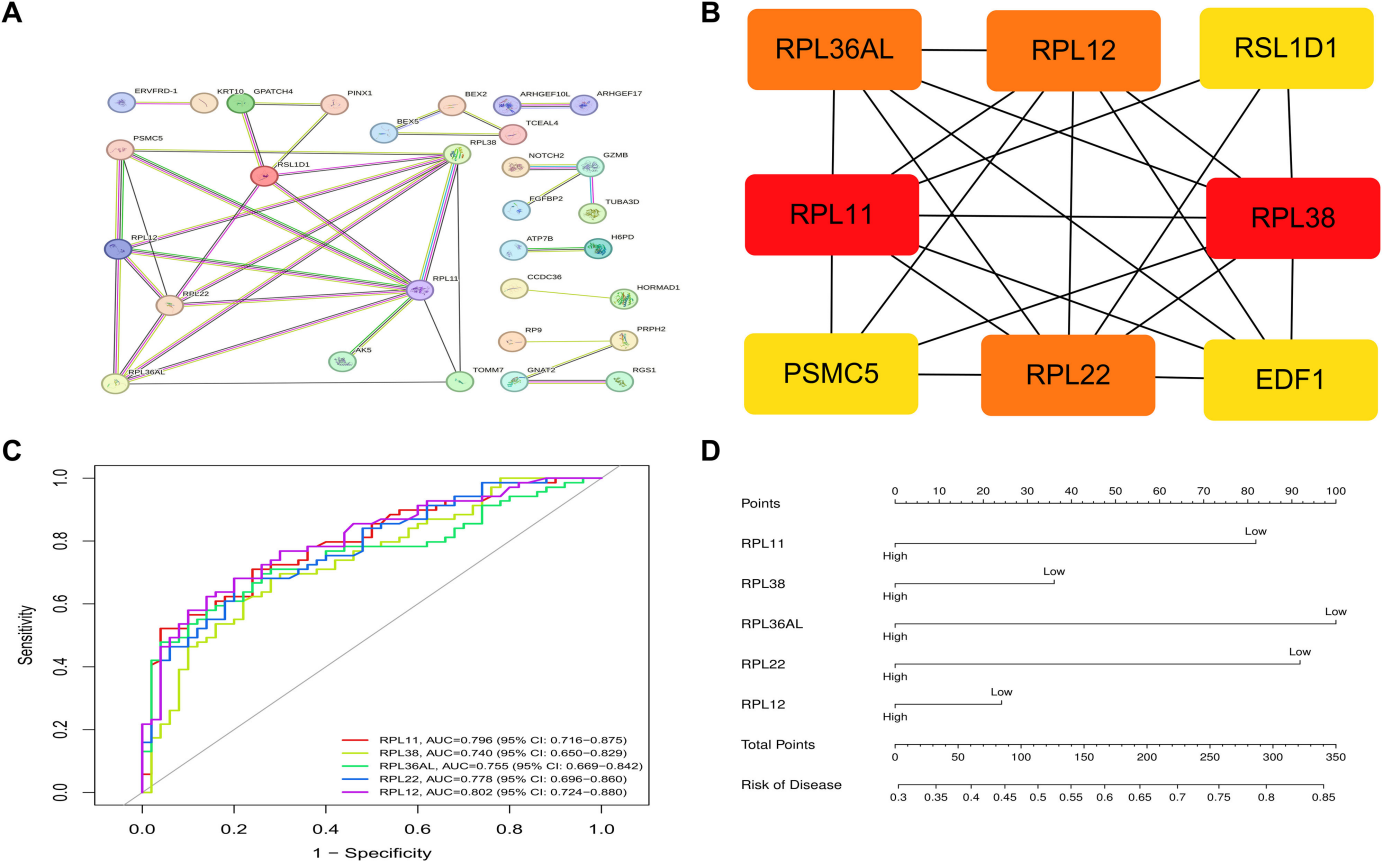
**(A)** PPI network. **(B)** Core gene network and ranking. **(C)** Receiver operator characteristic (ROC) curve. **(D)** Nomogram for predicting.

We developed a protein–protein interaction (PPI) network for the 201 DR key relevant genes via the STRING online database (confidence score ≥ 0.4), to construct DR-specific protein interaction patterns. The TSV-format data were imported into Cytoscape software(v.3.10.3) for visualization and analysis. By employing the CytoHubba plug-in alongside the degree algorithm, we effectively identified key genes, which were ranked on the basis of their connectivity from highest to lowest as follows: RPL11 (LogFC=-0.67, adjusted P = 4.19×10^-5^), RPL38 (LogFC=-0.67, adjusted P = 0.004), RPL12 (LogFC=-0.64,adjusted P = 2.85×10^-5^), RPL22 (LogFC=-0.64, adjusted P = 0.0002) and RPL36AL (LogFC=-0.65, adjusted P = 0.0012) (**Figures 6A, B**).

### Core gene validation

The five pivotal genes, namely, RPL12, RPL11, RPL22, RPL36AL, and RPL38, demonstrated significant diagnostic potential in differentiating DR samples from Nor. The corresponding AUC values are 0.802 (P < 0.001), 0.796 (P < 0.001), 0.778 (P < 0.001), 0.755 (P < 0.001), and 0.740 (P < 0.001), all of which surpass the threshold of 0.74 (**Figure 6C**). This finding indicates robust sensitivity and specificity, thereby reinforcing the validity of these core genes as viable target genes.

Additionally, a nomogram was constructed via the rms R package (version 8.1-0) to predict DR risk based on the five core genes. A comparison between the actual occurrence rates and the probabilities forecasted by the nomogram and decision curve analysis (DCA) revealed that our model displayed favorable predictive performance. (**Figure 6D**; **Supplementary Figure 3**).

### Role of immune cells in DR

**Figure 7:**
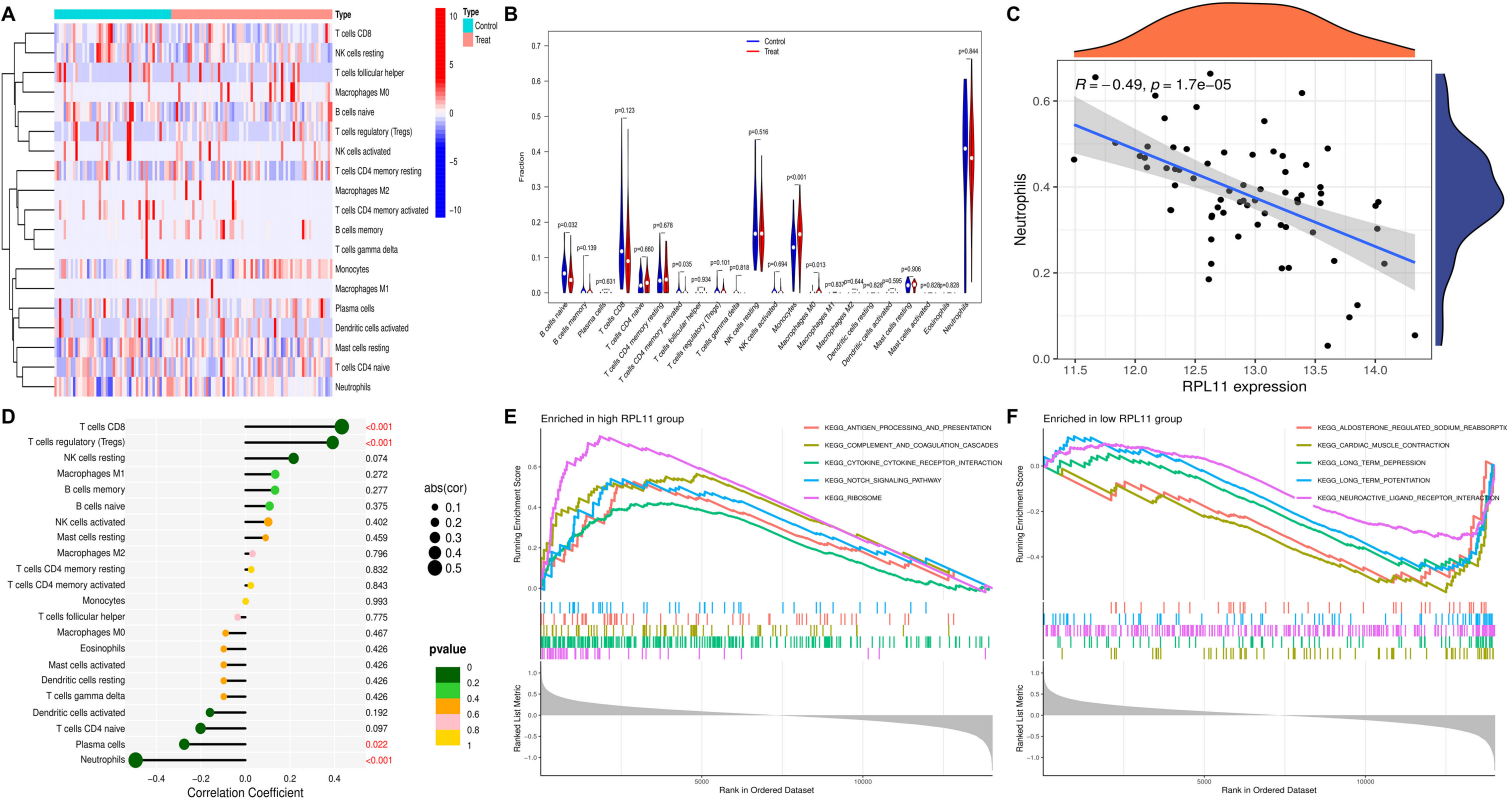
**(A)** immunocyte thermogram. **(B)** Immune cell differential analysis. **(C)** RPL11 is related to immunocyte infiltration levels. Correlation map illustrating the relationship between RPL11 expression and Neutrophils. **(D)** Lollipop plot visualizing the correlation between RPL11 and immune cells. **(E)** The top 5 GSEA enrichment pathways up-regulated in RPL11. **(F)** The top 5 GSEA enrichment pathways down-regulated in RPL11.

The analysis of functional and pathway interactions among co-expressed genes in DR revealed a notable association between DR and processes related to inflammation and the immune response. To assess the characteristics of immune cells, we utilized the CIBERSORT algorithm(version 1.03), which facilitates an examination of the relationship between co-expressed genes in DR and the infiltration of immune cells. The distribution of a panel of different immune cell types present in each sample is illustrated in **Figure 7A**. Significant differences were observed in B-cell naive (P=0.036), activated memory CD4^+^ T cells (P=0.031), monocytes (P=0.001) and M0 macrophages (P=0.009) between DR and control samples (**Figure 7B**).

### RPL11 genes in immune cell infiltration

CIBERSORT analysis revealed statistically significant correlations for RPL11, with negative regulation with neutrophils (R=-0.49, P=1.7E-05) and plasma cells (R=-0.27, P=0.022) and positive associations with CD8^+^ T cells (R=0.43, P=2E-04) and regulatory T cells (R=0.39, P=0.00087) (**Figures 7C, D**; **Supplementary Figures 4**-**6**). This study revealed a significant positive association between RPL11 co-expression patterns and increased immune cell infiltration in DR patients, suggesting that these ribosomal proteins may be associated with immunomodulatory effects via potential interactions with antigen presentation pathways and cytokine signaling cascades in the retinal vascular microenvironment.

### GSEA

The findings indicated that RPL11 was enriched in 47 KEGG signaling pathways via GSEA (fgsea R package, version 1.35.8). GSEA revealed divergent pathway activation profiles between RPL11 high-expression (≥75th percentile) and low-expression (≤25th percentile) subgroups. The five most significantly enriched KEGG pathways (ranked by *P* value) were identified in both cohorts. The high-RPL11 cohort demonstrated significant enrichment of KEGG_RIBOSOME (NES = 2.79, P = 1.82×10^-8^), KEGG_COMPLEMENT_AND_COAGULATION_CASCADES (NES = 2.22, P = 4.50×10^-5^), and KEGG_ANTIGEN_PROCESSIN-G_AND_PRESENTATION (NES = 2.02, P = 0.002), indicating enhanced protein synthesis, inflammatory cascades, and immune activation. Additionally, KEGG_NOTCH_SIGNALING_PATHW-AY (NES = 1.91, P = 0.005) and KEGG_CYTOKINE_CYTOKINE_RECEPTOR_INTERACTION (NES = 2.03, P = 2.98×10^-7^) were upregulated, suggesting dysregulated cellular differentiation and proinflammatory signaling (**Figure 7E**). In contrast, the low-RPL11 group was enriched in KEGG_CARDIAC_MUSCLE_CON-TRACTION (NES = -2.13, P = 4.74×10^-5^), KEGG_ALDOSTERON-E_REGULATED_SODIUM_REABSORPTION (NES = -1.74, P = 0.017), and synaptic plasticity-related pathways, including KEGG_LONG_TERM_DEPRESSION (NES = -1.74, P = 0.011) and KEGG_LONG_TERM_POTENTIATION (NES = -1.76, P = 0.011), indicating metabolic imbalance and neuronal signaling perturbations. KEGG_NEUROACTIVE_LIGAND_RECEPTOR_I-NTERACTION (NES = -1.51, P = 0.011) further highlighted aberrant neuromodulatory mechanisms, leading to edge analysis of the ribosome pathway (**Figure 7F**).

### RPL11 ligand molecular docking

**Figure 8:**
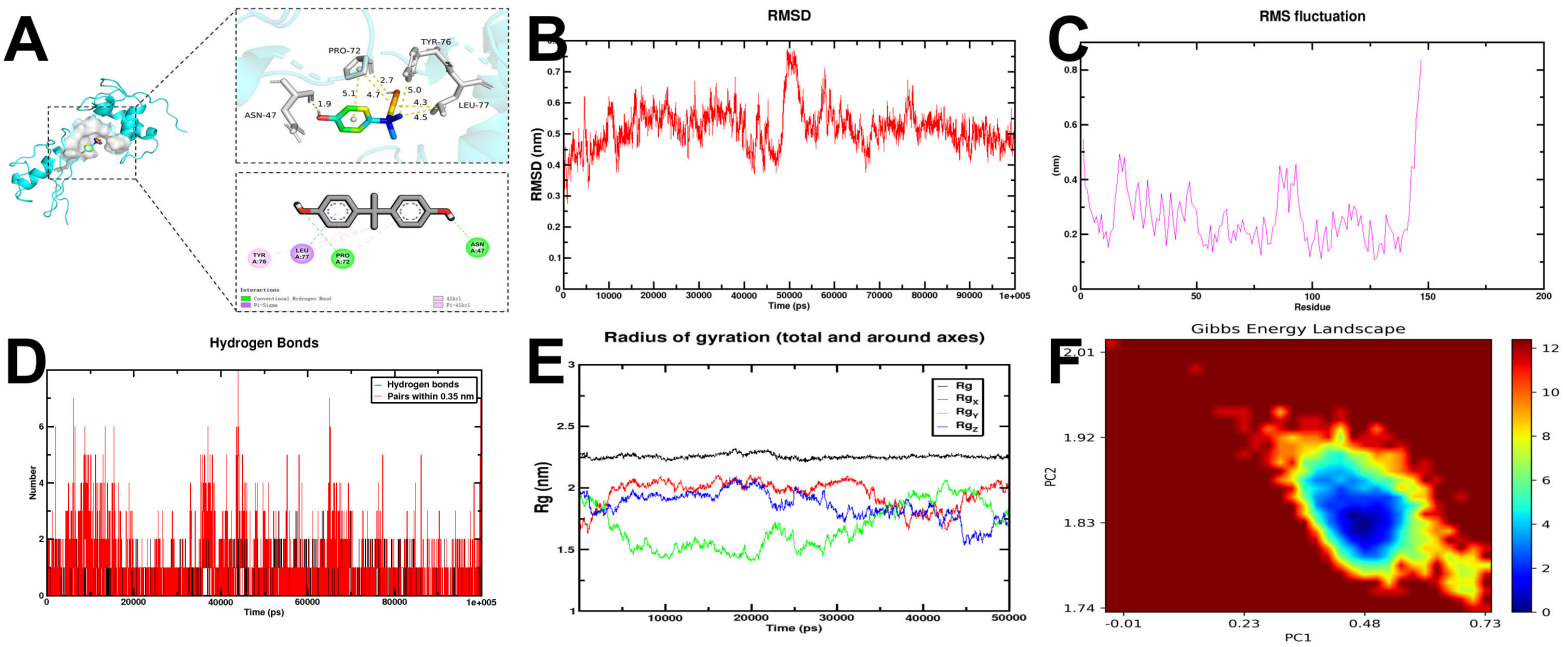
**(A)** Molecular docking analysis: BPA was docked with RPL11. **(B)** Molecular dynamics simulation analysis of the BPA-RPL11 complex. Root mean square deviation (RMSD) of the complex. **(C)** Root mean square fluctuation (RMSF) of the complex. **(D)** Hydrogen bonds of the complex. **(E)** The radius of gyration (RG) and its values along the three axes (Rgx, Rgy, Rgz). **(F)** Two-dimensional Gibbs free energy landscape of the complex.

Using the Comparative Toxicogenomics Database (CTD), we identified bisphenol A (BPA) as a high-probability interactor (inference score ≥20) for RPL11 through curated chemical–gene interaction filters. The compound CID of bisphenol A (6623) was identified through PubChem database queries. The three-dimensional crystal structure of RPL11 corresponding to PDB ID 2H8W was subsequently retrieved from the Protein Data Bank (PDB). The relevant structural files were retrieved and downloaded for computational analysis. Molecular docking simulations were performed via AutoDock Vina (version 1.2.0) to evaluate ligand–protein interactions. Notably, BPA exhibited a binding energy of -5.491 kcal/mol—while slightly higher than the -7 kcal·mol^-^¹ threshold, this value is significantly lower than that of negative control compounds (e.g., inactive BPA analogs, average ΔG = -2.1 ± 0.3 kcal·mol^-^¹), confirming its specific affinity for RPL11. This binding profile indicates BPA is a potential RPL11-interacting factor linked to DR risk. Molecular interaction patterns were subsequently visualized through three-dimensional structural representations using PyMOL (version 2.5.2) (**Figure 8A**).

### Molecular dynamics simulation

The BPA-RPL11 complexes exhibited stable conformational dynamics over the 100 ns simulation, with the backbone RMSD stabilizing at 0.45–0.55 nm from 60 ns onward (**Figure 8B**). These values suggest high structural persistence of the complex under physiological conditions. The RMSF curve revealed slight variations in the protein, suggesting a stable conformation of its domains and preservation of functional regions (**Figure 8C**). Time-dependent hydrogen bond analysis revealed persistent interactions between BPA and RPL11, with up to 5 hydrogen bonds formed at peak occupancy (**Figure 8D**). The establishment of hydrogen bonds between the ligand and the receptor contributed to the stabilization of the complex. The SASA of the complexes indicated enhanced hydrophobic burial. Moreover, the Rg of RPL11-BPA complexes persisted consistently within the range of 1.5–2.25 nm (**Figure 8E**), suggesting minimal global conformational changes upon BPA binding. Additionally, the Rg curve exhibited a steady degree of compactness during the entire simulation, thereby underscoring the structural stability of the complex. **Figure 8F** illustrates the two-dimensional Gibbs energy landscapes corresponding to the RPL11-BPA complexes. The complexes demonstrated a reduced Gibbs free energy when the Rg values ranged from 1.79 to 1.88 nm, and the RMSD values fell between 0.35 and 0.54 nm.

## Discussion

DR remains a leading cause of irreversible blindness in working-age adults globally, with its pathogenesis underpinned by intricate crosstalk between metabolic dysregulation, microvascular injury, and chronic inflammation (10). Despite advancements in therapeutic interventions such as anti-VEGF agents and laser photocoagulation, the subclinical progression and limited early diagnostic tools necessitate a deeper understanding of novel pathogenic mechanisms (11). Emerging evidence highlights environmental endocrine-disrupting chemicals (EDCs) as underappreciated risk factors for metabolic complications, yet their role in ocular microangiopathies such as DR remains largely unexplored.

BPA, a ubiquitous EDC with documented metabolic disrupting effects, has been identified in 93% of human biological samples, raising concerns about its potential involvement in diabetic complications (12). Against this backdrop, our study integrated transcriptomic profiling, WGCNA and computational toxicology to identify RPL11 as a pivotal molecular target in DR, with a focus on its interaction with BPA. Herein, we contextualize our findings within the literature, expound the mechanistic implications and discuss their clinical significance.

Our analysis revealed 341 DEGs in DR, with a predominance of upregulated transcripts (232 vs. 109 downregulated), reflecting the complex transcriptional reprogramming underlying retinal microvascular dysfunction. WGCNA further clustered genes into 38 modules, with the “black” and “brown” modules exhibiting the strongest correlation with DR pathogenesis. The 201 overlapping genes between DEGs and module core genes were enriched in pathways critical to cellular homeostasis, including response to light intensity (GO-BP) and cytosolic large ribosomal subunit (GO-CC). These findings align with prior reports implicating chromosomal instability and ribosomal dysfunction in diabetic microangiopathies, where hyperglycemia-induced oxidative stress disrupts genomic integrity and protein synthesis machinery (13). Notably, ribosomal pathways emerged as a central hub, with GO analysis highlighting the “structural constituent of ribosome” as a key molecular function. These findings underscore the potential role of ribosomal protein dysregulation in DR, where impaired translation efficiency may exacerbate retinal cell stress and inflammation (14).

Among the core genes identified, RPL11 stood out for its robust diagnostic performance in peripheral blood (AUC = 0.796, 95% CI:0.716-0.875) and consistent downregulation in DR- related peripheral blood samples. As a component of the 60S ribosomal subunit, RPL11 is essential for ribosome assembly and translational fidelity in cellular processes (15). Its downregulation in DR-related peripheral blood samples was associated with ribosomal function-related pathways in our analysis, which echoes observations in other diabetic complications where ribosomal stress is linked to apoptosis and proinflammatory cytokine release—though direct relevance to retinal ribosomal dysfunction requires further validation (16). The diagnostic utility of RPL11 in peripheral blood is further supported by its high specificity and sensitivity, outperforming other ribosomal proteins (e.g., RPL38, AUC = 0.740, 95% CI:0.650-0.829) in the same peripheral blood dataset. These findings align with recent studies identifying ribosomal proteins as potential peripheral blood biomarkers in metabolic diseases, including diabetic nephropathy (17). Collectively, these data position RPL11 as a hypothesis-generating candidate for early detection of DR based on peripheral blood biomarkers, particularly in asymptomatic stages where traditional screening methods fail– with its relevance to retinal tissue requiring additional research.

Our immune infiltration analysis revealed significant perturbations in DR, with increased proportions of CD4^+^ memory-activated T cells, monocytes, and M0 macrophages—cell types linked to retinal inflammation and angiogenesis (18, 19). Notably, RPL11 expression was inversely correlated with neutrophils and plasma cells but positively correlated with CD8^+^ T cells and regulatory T cells. This pattern suggests that RPL11 may modulate immune homeostasis in DR: CD8^+^ T cells contribute to cytotoxic responses against stressed retinal cells, whereas Tregs suppress excessive inflammation (20). The inverse correlation between RPL11 and neutrophils is particularly intriguing, as neutrophil extracellular traps (NETs) have been implicated in DR-associated vascular damage (21). Reduced RPL11 may thus exacerbate NET formation, further promoting microvascular injury. These findings expand our understanding of DR beyond metabolic dysfunction to include immune dysregulation, with RPL11 serving as a potential link between ribosomal stress and immune imbalance.

Our observation of altered immune cell populations aligns with the established literature: M0 macrophages polarize to proinflammatory M1 phenotypes in DR, contributing to vascular permeability (22, 23), whereas CD4^+^ memory T cells drive chronic inflammation (24). The association between RPL11 and Tregs further resonates with studies showing Treg dysfunction in DR (25), suggesting that RPL11 may preserve Treg-mediated immune tolerance. These findings reinforce the notion that DR is an immune-metabolic disorder in which ribosomal protein dysregulation intersects with immune cell dynamics to drive pathology.

Prior studies have linked ribosomal proteins to diabetic pathogenesis: RPL11 downregulation in pancreatic β-cells impairs insulin secretion (26–28), whereas ribosomal stress in endothelial cells exacerbates diabetic macroangiopathy (7). Our work extends this to DR, demonstrating that RPL11 deficiency is correlated with pathways enriched in nuclear chromosome segregation, suggesting potential genomic instability in retinal cells. Notably, RPL11 also interacts with MDM2 (mouse double minute 2 homolog), a tumor suppressor involved in cell cycle arrest and apoptosis, to regulate p53 activity (6). In DR, p53 activation contributes to retinal cell death (29), suggesting that RPL11 downregulation may dysregulate p53-mediated pathways, amplifying cellular damage.

BPA’s role in metabolic disorders is well established: it disrupts glucose homeostasis by impairing insulin sensitivity and β-cell function (4). Our novel finding—BPA’s binding affinity to RPL11 in computational simulations—represents a hypothesis-generating link that requires further validation to establish potential relevance to DR. Molecular dynamics simulations confirmed the stability of the BPA-RPL11 complexes under physiological conditions, with persistent hydrogen bonds and minimal conformational fluctuations, indicating a strong interaction in computational models. This interaction was predicted to potentially inhibit RPL11’s ribosomal function, which aligns with translational defect-related pathways observed in DR-related peripheral blood samples—though direct evidence of such inhibition in retinal tissues or DR pathogenesis is lacking and requires further study. BPA has previously been shown to disrupt protein–protein interactions in ribosomal complexes in non-ocular cell types (8), and our data suggest a potential similar interaction in computational models, with its relevance to ocular tissues remaining to be verified.

On the basis of our results, we propose a hypothetical model in which environmental BPA exposure may exacerbate DR through the following steps:1. BPA-RPL11 interaction: BPA binds to RPL11 with specific affinity, which may inhibit its ribosomal function (needs *in vitro* validation). 2.Ribosomal stress: Impaired ribosome assembly reduces translational efficiency, triggering cellular stress pathways. 3.Immune dysregulation: RPL11 downregulation shifts immune cell populations, promoting retinal inflammation. 4.Microvascular damage: The combined effects of ribosomal stress and immune imbalance exacerbate vascular leakage, neovascularization, and retinal degeneration (**Figure 1**).

The diagnostic performance of RPL11 supports its development as a noninvasive biomarker. A blood-based RPL11 assay could complement fundus photography for early DR detection, particularly in resource-limited settings. Therapeutically, targeting the BPA–RPL11 interaction (e.g., via small-molecule inhibitors) or restoring RPL11 expression may mitigate ribosomal stress and immune dysfunction in DR.

Given the ubiquity of BPA, reducing exposure could represent a cost-effective DR prevention strategy. Public health measures—such as regulating BPA in food packaging and promoting BPA-free alternatives—may provide a potential approach to reduce DR risk in diabetic populations (needs further validation). Healthcare providers should also counsel patients on minimizing BPA exposure, alongside traditional glycemic control.

Tissue specificity: Transcriptomic data derived from peripheral blood may not fully reflect retinal molecular changes, although circulating biomarkers often mirror tissue pathology (30). Confounder limitation: Individual-level data on age, sex, diabetes duration, HbA1c, and treatment status were not explicitly publicly available in the GSE221521 dataset. Molecular docking and simulations require validation *in vitro* (e.g., BPA-RPL11 binding assays) and *in vivo* (e.g., BPA-exposed diabetic mice).
Immune Mechanisms: CIBERSORT predictions need confirmation via flow cytometry or immunohistochemistry of retinal tissues.
Generalizability: The single dataset (GSE221521) limits broader applicability; replication in independent cohorts with complete confounder data is warranted.

Validation of the BPA–RPL11 interaction in retinal endothelial cells and DR animal models. The role of RPL11 in p53-mediated apoptosis and immune cell recruitment was explored via gene knockout/overexpression systems. Investigate other EDCs (e.g., phthalates) for interactions with DR-associated ribosomal proteins. Develop RPL11-targeted theranostics (e.g., dual diagnostic-therapeutic nanoparticles) for DR management.

## Conclusion

Our study identified RPL11 as a molecule with significant associations with DR in peripheral blood, where its downregulation is linked to ribosomal-related pathways, immune cell infiltration patterns (inferred from peripheral blood data), and environmental BPA exposure in computational analyses. The specific binding affinity of the BPA–RPL11 interaction observed in silico is a hypothesis-generating finding that may provide clues to potential environmental associations with DR, though further validation (including *in vitro*, *in vivo*, and retinal tissue-specific studies) is necessary to confirm its relevance to DR pathogenesis. The diagnostic potential of RPL11 in peripheral blood and the BPA–RPL11 axis as a candidate for further exploration offer hypothesis-generating directions to guide future DR research—definitive conclusions regarding any therapeutic or preventive value will require additional empirical evidence.

## Data Availability

The original contributions presented in the study are included in the article/**Supplementary Material**. Further inquiries can be directed to the corresponding author.

## Supplementary material

Supplementary Figure 1 Cross-module gene significance plot.

Supplementary Figure 2 The KEGG bubble map.

Supplementary Figure 3 Decision curve analysis (DCA).

Supplementary Figure 4 Correlation map illustrating the relationship between RPL11 expression and T cells regulatory.

Supplementary Figure 5 Correlation map illustrating the relationship between RPL11 expression and Plasma cells.

Supplementary Figure 6 Correlation map illustrating the relationship between RPL11 expression and T cells CD8.

Supplementary Figure 7 WGCNA softpower selection for choosing the most appropriate softpower value.
