# Supplementary figures and images for: Transcriptomic profiling of diabetic retinopathy: insights into RPL11 and bisphenol A
Source: Front Endocrinol (Lausanne). 2025 Nov 26;16:1705233. doi: 10.3389/fendo.2025.1705233 (PMC12689329; doi:10.3389/fendo.2025.1705233)

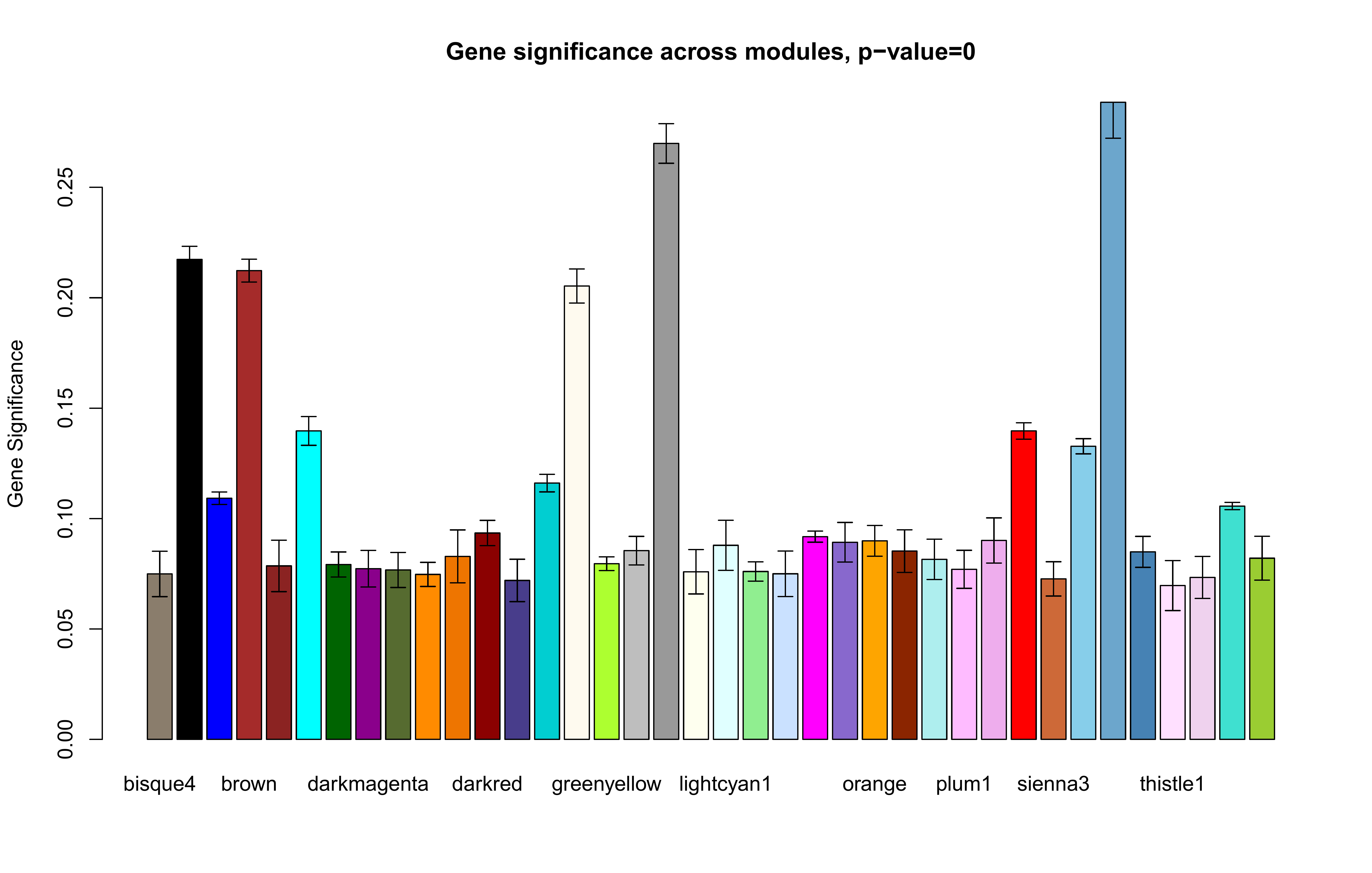

Supplement: Supplementary Figure 1 — Cross-module gene significance plot. [file Image1.jpeg]

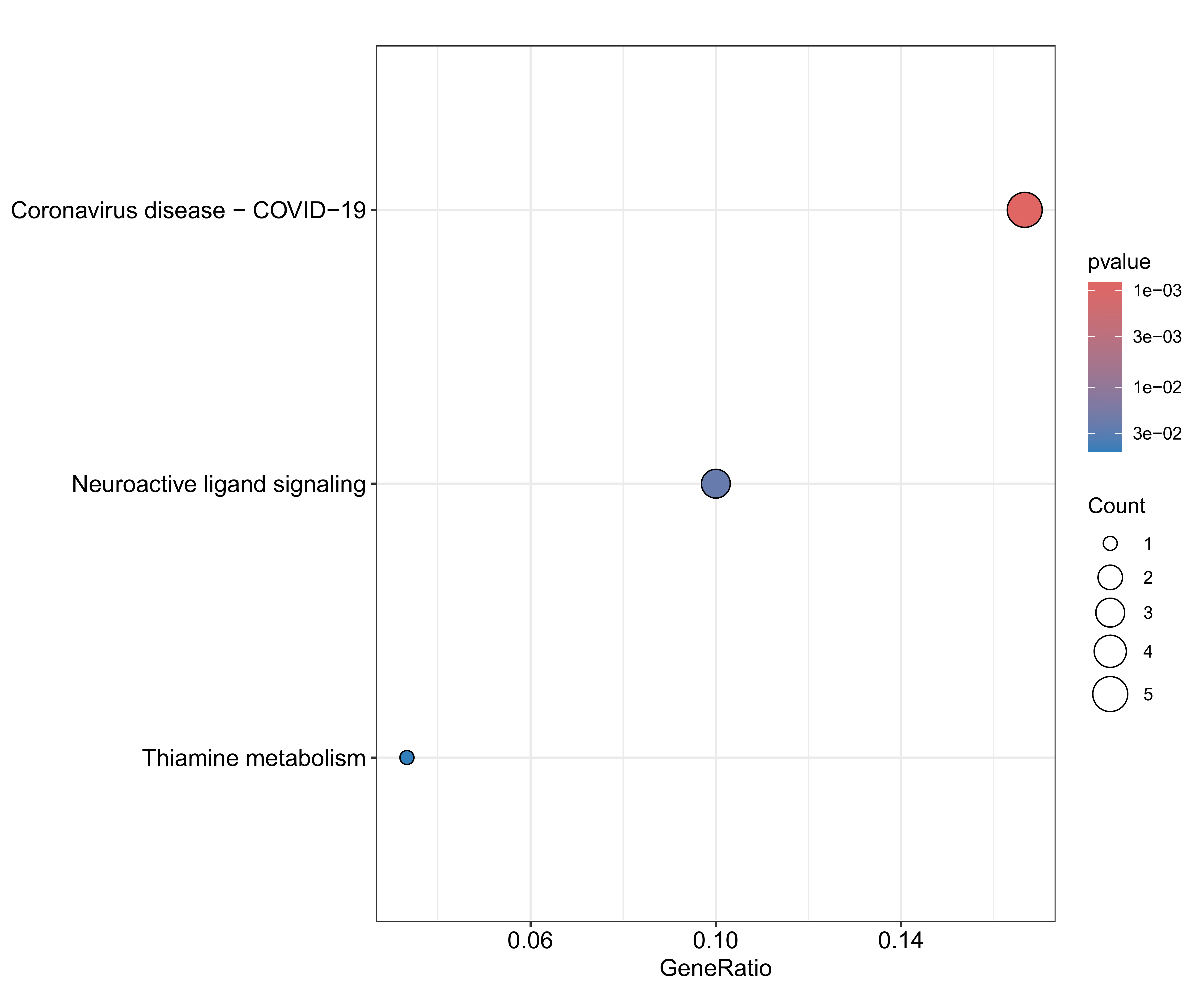

Supplement: Supplementary Figure 2 — The KEGG bubble map. [file Image2.jpeg]

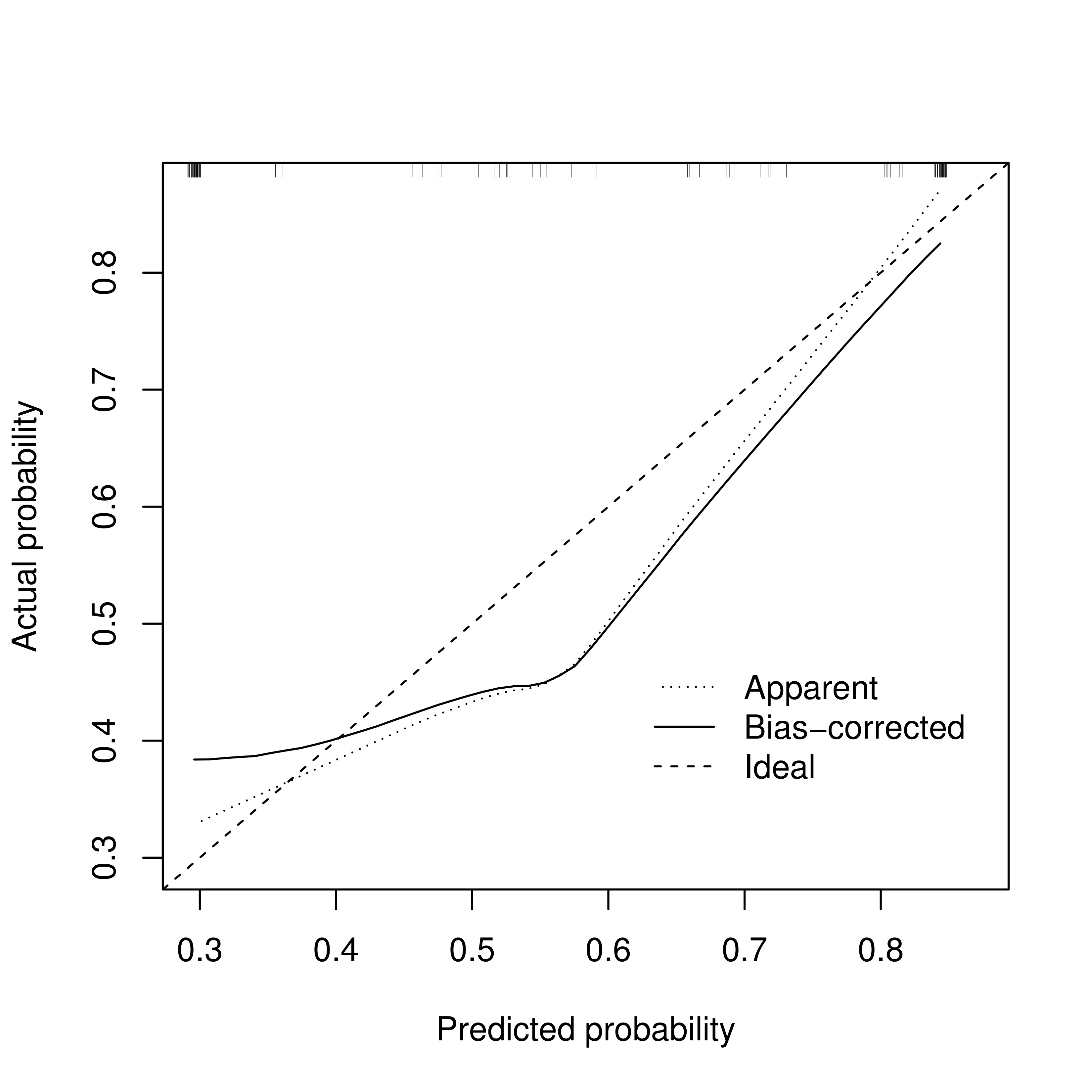

Supplement: Supplementary Figure 3 — Decision curve analysis (DCA). [file Image3.jpeg]

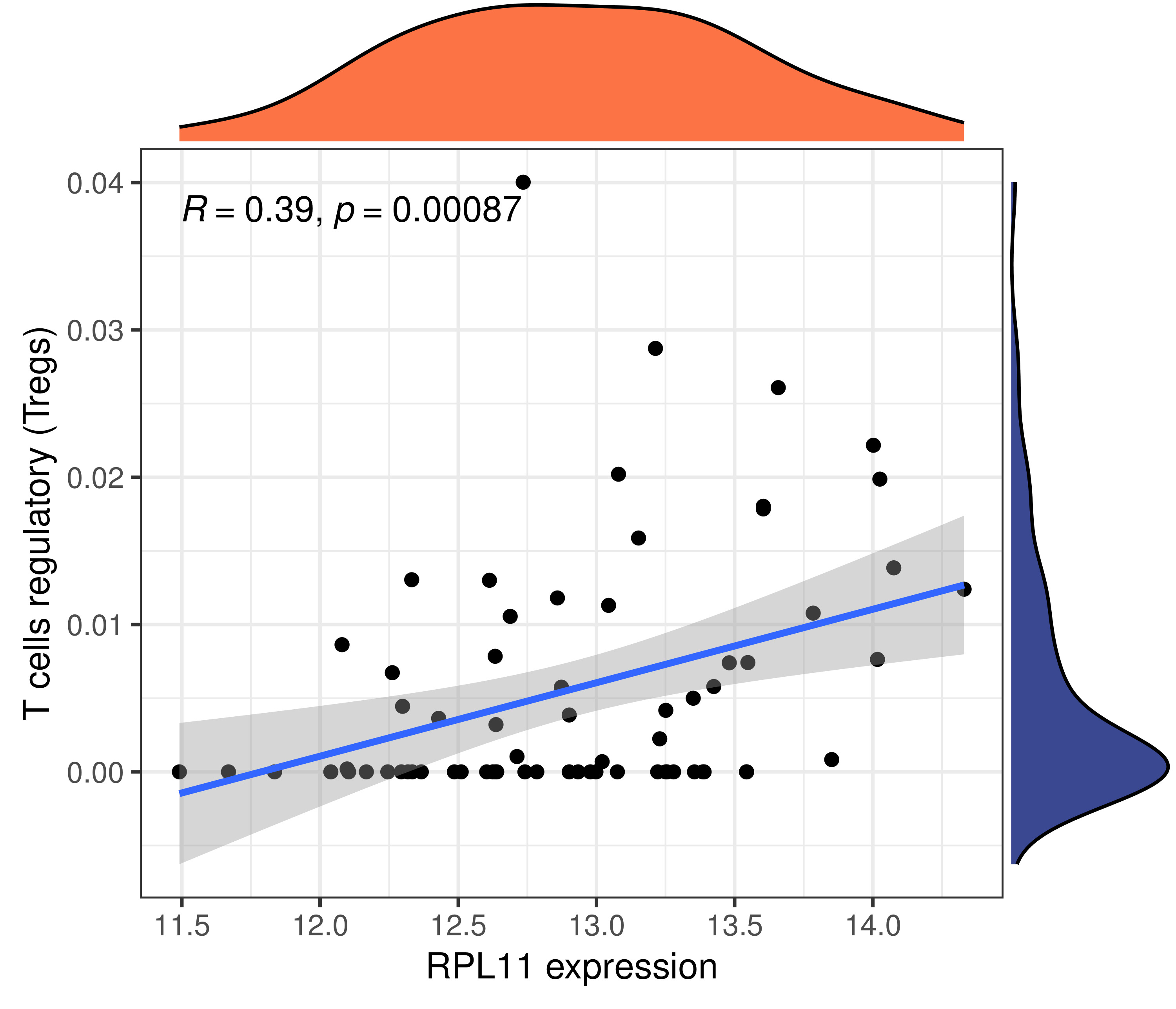

Supplement: Supplementary Figure 4 — Correlation map illustrating the relationship between RPL11 expression and T cells regulatory. [file Image4.jpeg]

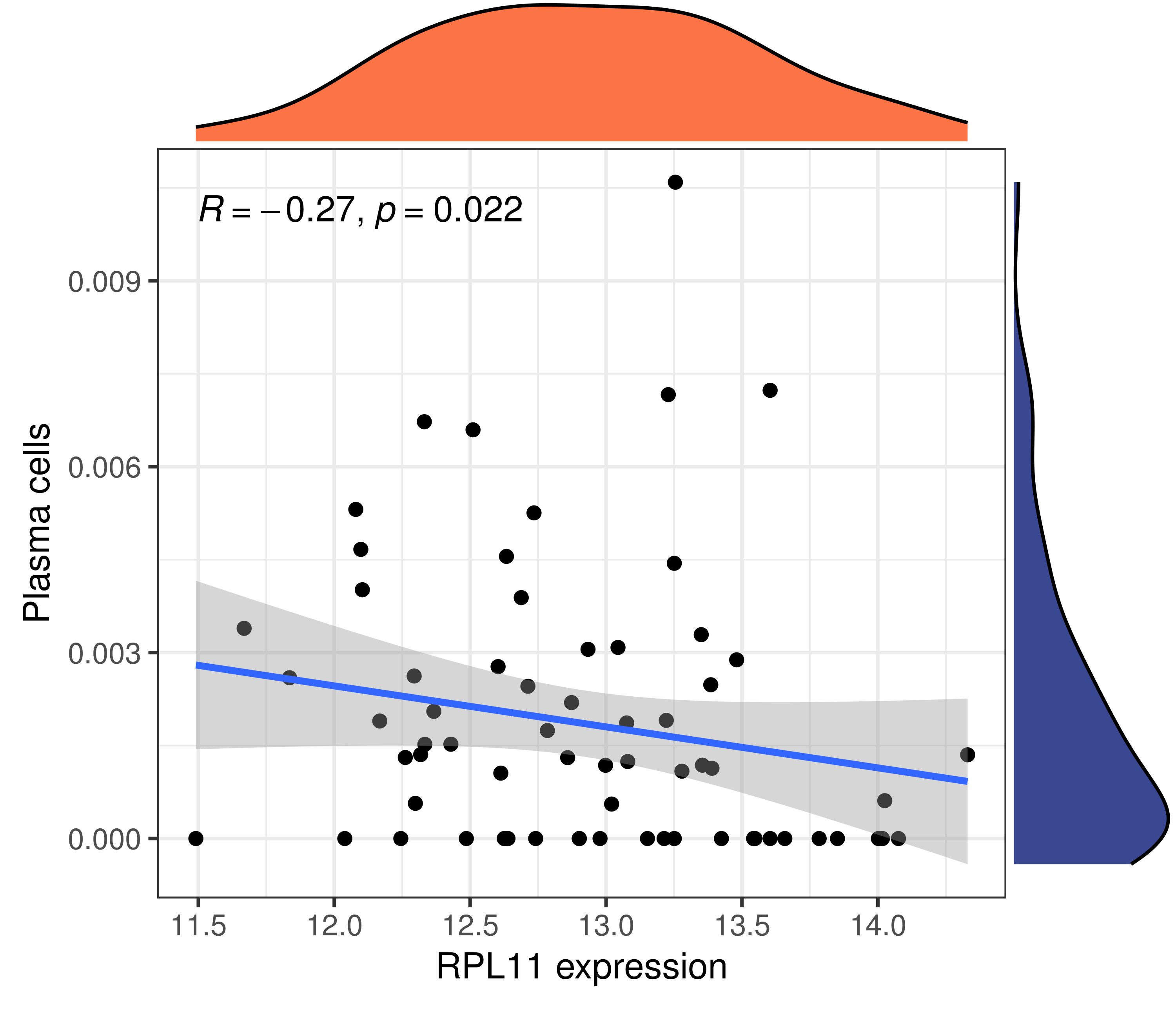

Supplement: Supplementary Figure 5 — Correlation map illustrating the relationship between RPL11 expression and Plasma cells. [file Image5.jpeg]

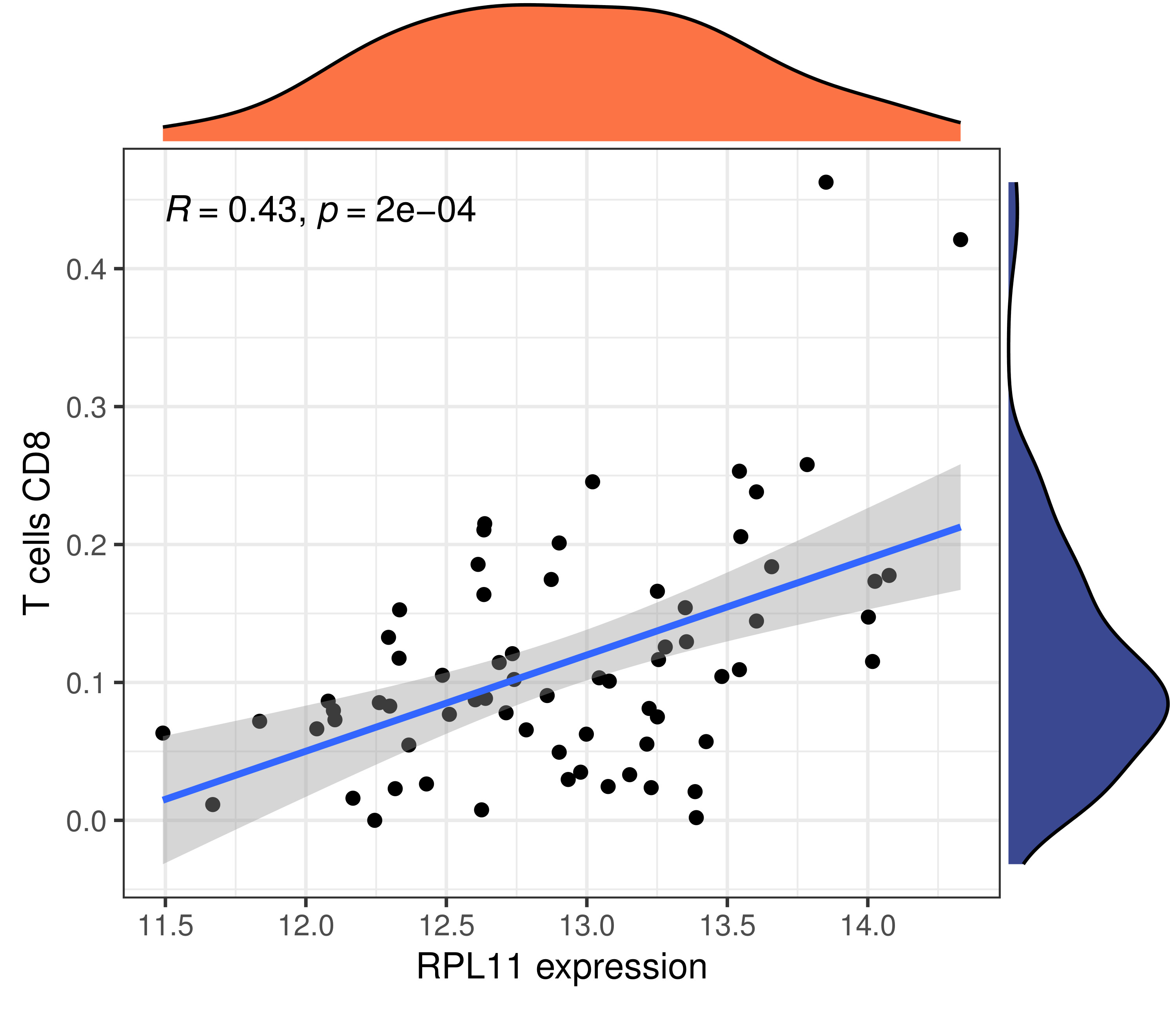

Supplement: Supplementary Figure 6 — Correlation map illustrating the relationship between RPL11 expression and T cells CD8. [file Image6.jpeg]

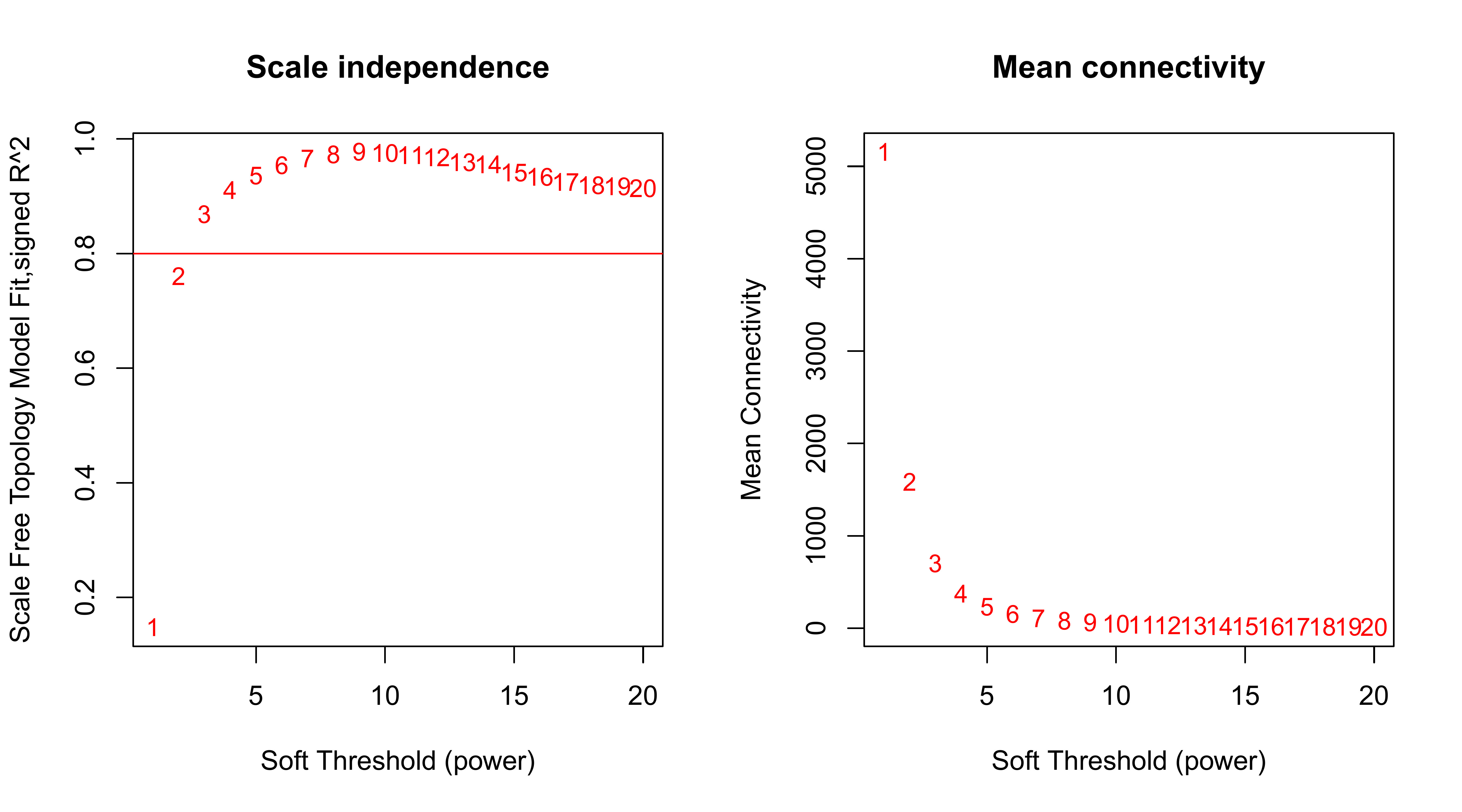

Supplement: Supplementary Figure 7 — WGCNA softpower selection for choosing the most appropriate softpower value. [file Image7.jpeg]
